# The relationship between antibiotic utilization for respiratory conditions (AXR) and antibiotic prescribing for pharyngitis

**DOI:** 10.1017/ash.2025.10129

**Published:** 2025-09-12

**Authors:** Divyam Goel, Meg Grimshaw, Nora Fino, Emily S. Spivak, Adam L. Hersh

**Affiliations:** 1 Division of Pediatric Infectious Diseases, Department of Pediatrics, Spencer Fox Eccles School of MedicineUniversity of Utah, Salt Lake City, UT, USA; 2 Division of Pediatric Infectious Diseases, Department of Pediatrics, Duke University School of Medicine, Durham, NC, USA; 3 Spencer Fox Eccles School of Medicine, University of Utah, Salt Lake City, UT, USA; 4 Divsion of Epidemiology, Department of Internal Medicine, Spencer Fox Eccles School of Medicine, University of Utah, Salt Lake City, UT, USA; 5 Division of Infectious Diseases, Department of Medicine, Spencer Fox Eccles School of Medicine, University of Utah, Salt Lake City, UT, USA

## Introduction

Urgent care (UC) is a priority for antibiotic stewardship, where upper respiratory infections (URIs) are a leading reason for care.^1^ Prescribing metrics for respiratory conditions are valuable to benchmark performance and identify areas for improvement. One new metric is antibiotic utilization for respiratory conditions (AXR), which calculates the percentage of all respiratory conditions where an antibiotic is prescribed.^2^ Although AXR provides insight into aggregate antibiotic use, its lack of specificity for prescribing by individual condition or appropriateness limits its direct application for specific stewardship interventions.^3^

Pharyngitis, accounts for the largest number of encounters and antibiotic prescriptions in our UC system.^4^ As part of a stewardship intervention, UC clinicians indicated that pharyngitis was a prominent area of antibiotic overuse, including empiric treatment without testing and treatment despite negative test results for group A Streptococcus (GAS). We used pharyngitis, a documented concern in our system, as a sample condition to explore the association between AXR and antibiotic overuse.

## Methods

This was a retrospective study of antibiotic prescribing for respiratory conditions in the University of Utah UC system from January 2019 to May 2024. This includes nine ambulatory clinics and telemedicine. Clinics use point-of-care molecular testing for GAS. This study was approved by the University of Utah Institutional Review Board.

We examined three metrics in aggregate and for individual clinicians (excluded if <100 encounters): (1) AXR, the percentage of respiratory diagnoses where an antibiotic was prescribed; (2) percentage of pharyngitis diagnoses with a negative GAS test with an antibiotic prescribed; and (3) percentage of pharyngitis diagnoses treated with an antibiotic that were not tested. To identify pharyngitis encounters, we used a classification system that assigns a single diagnosis for each encounter based on the diagnosis Tier using International Classification of Diseases (ICD) codes.^4,5^ We explored the relationship between AXR and prescribing for pharyngitis using Pearson’s correlation coefficient weighted by the number of encounters for each clinician.

Because some encounters had more than one diagnosis coded that could warrant an antibiotic (eg, pharyngitis and sinusitis), we randomly reviewed 100 UC encounters where the classification system assigned pharyngitis and an antibiotic was prescribed, and either GAS testing was not performed or was negative.

## Results

Of 100 pharyngitis encounters reviewed, 72 were treated despite a negative test and 28 were treated with no GAS test. Of these, we identified 26 where an alternate diagnosis was responsible for an antibiotic prescription based on clinician documentation, including otitis media and sinusitis. For 21, ICD codes were present for these conditions. Therefore, to improve validity, for our subsequent analysis of metrics related to treatment of pharyngitis, we excluded encounters with these codes present.^5^ No cases were empiric treatment for peritonsillar abscess.

**Figure 1.**
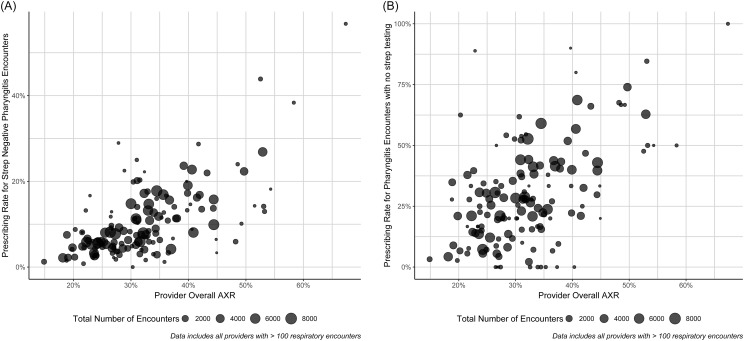
Overall prescribing by clinicians compared to their prescribing rate for strep-negative pharyngitis encounters [**Panel A**] and pharyngitis encounters with no strep testing [**Panel B**]. The size of each point represents the total number of respiratory encounters (of any kind, not just pharyngitis) over the study period; only providers with at least 100 respiratory encounters are shown. Pharyngitis encounters with an additional diagnosis of otitis, sinusitis, otalgia, or bronchitis are excluded.

After exclusions, there were 339,119 respiratory encounters (mean age 30 yrs, 58% female). 150 clinicians had >100 encounters and contributed to the analysis. The aggregate AXR was 31.6%. The prescribing rate for GAS test-negative pharyngitis encounters was 9.7% (provider range 0%, 56.76%). The prescribing rate for pharyngitis encounters without GAS testing was 29.2% (provider range 0%, 100%). When AXR was examined compared to the antibiotic prescribing rate for each pharyngitis metric (Figure 1), moderate correlations were observed for each group (*R* ^2^ = .49, .40 for test negative and no test, respectively).

## Discussion

We explored the relationship between AXR and specific prescribing metrics for pharyngitis in our UC system. We found the validity of pharyngitis as the actual diagnosis responsible for an antibiotic prescription in our system is suboptimal, even when using a commonly applied classification system without excluding concomitant diagnoses for common conditions (eg, sinusitis). Second, we found moderate association for individual clinicians between AXR and inappropriate prescribing for pharyngitis.

Ensuring appropriate antibiotic prescribing for GAS pharyngitis is a priority in our system since this is the leading reason for antibiotics in UC. In addition to ensuring treatment is supported by testing and not empiric, we also focused on overtreatment in instances where testing was negative, which has been a focus in prior studies.^6^ AXR, a measure of aggregate antibiotic use, does not account for the specific nature of inappropriate prescribing such as the metrics for pharyngitis we considered. It similarly does not account for other dimensions of stewardship, including extended treatment duration and incorrect antibiotic choice, which may be patterns of concern in other UC systems. Our exploration of AXR and specific metrics for pharyngitis indicates the utility of using AXR as a high-level screening metric among clinicians to signal a tendency for inappropriate prescribing for pharyngitis. The extension of this application to other specific cases of inappropriate antibiotic use deserves study.

Although the Tier system for classifying diagnoses in outpatient encounters is a useful method for linking antibiotic prescriptions to the intended diagnosis when multiple diagnoses are assigned, there has been limited validation. In the case of pharyngitis, our findings suggest there is potential for misclassification when multiple diagnoses are present that may warrant antibiotics. The accuracy of pharyngitis diagnosis is improved if encounters with these concomitant diagnoses are excluded.

Limitations to this study include performance in one setting and uncertainty about application to other systems. Some patients may have had GAS testing performed before the encounter that was not captured.

We found variability in prescribing metrics for pharyngitis, with higher rates of inappropriate prescribing associated with higher prescribing overall. AXR may be a useful screening metric for inappropriate prescribing, allowing further exploration into diagnostic and prescribing practices for pharyngitis, tailored education, and intervention.^3^
